# Immunoexpression profile of LATS2 and YAP1 and its clinicopathological relevance in oral tongue squamous cell carcinoma

**DOI:** 10.1007/s10006-026-01557-1

**Published:** 2026-04-14

**Authors:** Ondina Karla Mousinho Rocha Torres, André Luis Alves Borges, Lucas Melo da Costa, Débora Frota Colares, Éricka Janine Dantas da Silveira, Márcia Cristina Costa da Miguel

**Affiliations:** 1 Postgraduate Program in Dental Sciences, Department of Oral Pathology, Federal University of Rio Grande do Norte, Natal, Rio Grande do Norte Brazil; 2 Department of Oral Pathology, Federal University of Rio Grande do Norte, Natal, Rio Grande do Norte Brazil

**Keywords:** Hippo pathway, Oral squamous cell carcinoma, Immunohistochemical, Prognosis, Tongue neoplasms

## Abstract

**Objective:**

The objective of this study was to investigate the immunohistochemical expression of the Hippo pathway proteins LATS2 and YAP1 in oral tongue squamous cell carcinomas (OTSCCs) and normal oral mucosa (NOM), as well as potential relationships between them and clinicopathological characteristics and survival outcomes.

**Materials and methods:**

Twenty-six cases of OTSCC and 8 samples of NOM were analyzed. Clinical data were obtained from medical records. Morphological assessment was based on the WHO (2005) grading system and the combined score of tumor cell nests (B) and tumor invasion depth (D) (BD model). Immunoexpression of LATS2 and YAP1 was determined by immunohistochemistry.

**Results:**

In NOMs, LATS2 expression was observed across all epithelial layers, with nuclear predominance, whereas YAP1 was observed in the basal and parabasal layers. YAP1 expression was significantly higher in OTSCCs (*p* < 0.001) and was significantly associated with the high-risk BD model (*p* = 0.034). OTSCCs with reduced LATS2 immunoexpression (*p* = 0.042) had poorer disease-free survival (DFS), while well-differentiated cases according to WHO presented better DFS outcome (*p* = 0.022). Multivariate analysis identified low LATS2 expression (HR 0.19; 95% CI 0.05–0.75; *p* = 0.017) and tumor size (HR 3.93; 95% CI 1.02–15.06; *p* = 0.046) as independent prognostic factors for 5-year DFS in OTSCC.

**Conclusion:**

The functional alterations of LATS2 and YAP1 may reflect the dysregulation of the Hippo pathway in OTSCC, which possibly influences mechanisms of tumor progression and clinical behavior in this carcinoma.

**Supplementary Information:**

The online version contains supplementary material available at 10.1007/s10006-026-01557-1.

## Introduction

Occupying the sixteenth position worldwide in incidence and mortality rates, oral squamous cell carcinoma (OSCC) is a frequent malignancy in the head and neck region, characterized by aggressive growth and originates from the squamous epithelium of the oral cavity [1]. Among the various affected sites, such as the floor of the mouth, hard palate, and lip, the tongue represents a significant portion, accounting for approximately 31.9% of all cases in the oral cavity [1, 2].

Oral tongue squamous cell carcinoma (OTSCC) is biologically distinct, and it is considered an aggressive subtype that exhibits a high potential for local invasion and lymph node metastasis [3]. The prognosis for this condition is challenging, even when diagnosed in early stages. The low 5-year overall survival (OS) rates may indicate a distinct molecular behavior from other carcinomas arising in other head and neck subsites [3, 4].

Thus, research efforts have been focused on discovering new methods for early detection and risk assessment to identify factors that can predict disease progression and tumor behavior and therefore guide therapeutic modalities and improve patient prognosis [5, 6]. In this context, the role of the Hippo pathway is highlighted, which is involved in controlling organ size, tissue homeostasis, and regeneration, but which, when dysregulated, influences the process of carcinogenesis [7].

The Hippo signaling pathway consists of a wide range of proteins acting in a cascade [8]. In summary, this pathway begins with a core kinase cascade, involving a pair of serine/threonine kinases, MST1/2, which then induces the activation of LATS1/2. These, consequently, lead to the phosphorylation of the transcriptional coactivators YAP1 and TAZ, which bind to a protein called 14-3-3, preventing them from remaining in the nucleus and keeping them retained in the cytoplasm, where they are polyubiquitinated and degraded, thus preventing gene activation and cell growth, making the Hippo pathway a tumor suppressor [9].

Conversely, when YAP1 and TAZ are not phosphorylated, they are translocated to the nucleus, playing a pro-oncogenic role, as they can interact with TEAD and positively modulate the expression of target genes related to cell proliferation, in addition to inhibiting apoptosis and contributing to the migration, invasion, and transformation of tumor cells [9, 10].

Although the Hippo pathway has been associated with multiple events in the process of carcinogenesis, its role is not yet completely elucidated. The expression of its components can contribute to the evolution of various types of malignant tumors, acting as prognostic indicators, as observed in head and neck carcinomas [10]. This study analyzed the expression of the proteins LATS2 and YAP1, involved in Hippo pathway signaling, in OTSCCs and investigated their relationship to clinicopathological and survival parameters.

## Materials and methods

### Study design

The research was approved by the Research Ethics Committee of the Federal University of Rio Grande do Norte (UFRN) (Approval No. 5,288,936). Twenty-six cases of OTSCC and 8 specimens of normal oral mucosa (NOM) were included, obtained from perilesional areas of patients who underwent surgical treatment for the removal of benign lesions and diagnosed at the UFRN Oral Pathology Service by an experienced pathologist. The perilesional regions showed no epithelial alterations or inflammatory infiltrate in the underlying connective tissue.Clinical information was collected from the clinical records of these patients. Cases that received chemotherapy and/or radiotherapy prior to surgical treatment were excluded. For survival analysis, only cases with a minimum follow-up of 60 months were included. The outcomes analyzed were OS, disease-specific survival (DSS), and disease-free survival (DFS).

### Histomorphological analysis

The slides stained by the hematoxylin and eosin technique for each specimen were evaluated and classified according to the criteria proposed by the WHO (2005) grading system and the BD model by Almangush et al. (2014) [11, 12]. This analysis was performed at different times by two previously trained examiners (MCCM and OKMRT). In cases of inter-examiner divergence, the cases were re-evaluated to reach a consensus.

### Immunohistochemical study

All paraffin-embedded specimens were sectioned into 3 μm slices and subjected to deparaffinization, rehydration, and antigen retrieval with the Trilogy solution (Cell Marque, USA). After blocking endogenous peroxidase and washing, the slides were incubated with a protein blocker (ThermoScientific, UK) and washed with Tris buffer (Sigma Chemical, USA; pH 7.4). Next, they were incubated with the primary antibodies anti-LATS2 (Invitrogen^®^, 1:500; overnight) and anti-YAP1 (Clone 1A12; Cell Signaling Technology^®^, 1:600; 60 min). Subsequently, incubation was performed with the HiDef system (Cell Marque, USA) for 60 min, followed by development with diaminobenzidine (DAB), counterstaining with Mayer’s hematoxylin, and mounting with Permount^®^ resin (Fisher Scientific, USA). For the negative control, 1% BSA in buffer solution was used to substitute the primary antibody, while the positive control was the perilesional epithelium, due to the known immunopositivity of LATS2 and YAP1 in the basal cells of the oral mucosa.

### Analysis of the immunohistochemical profile

After processing, the immunohistochemical analysis was performed independently by two previously trained and calibrated examiners, who were blinded to the clinical and histomorphological data. In cases of scoring disagreement, the slides were re-examined conjointly by both observers until a final consensus was reached to ensure diagnostic consistency.

The immunoexpression of LATS2 and YAP1 in LSCCs and normal oral mucosa was evaluated based on the methodology proposed by Ono et al. (2019) [13]. Brown staining in the nucleus and/or cytoplasm throughout the epithelium was considered positive, and the analyses were conducted through light microscopy at 40x, 100x, and 400x magnifications. Immunoexpression was classified on a scoring scale from 0 to 3: score 0 indicated absence of staining or positive cells only in the basal and parabasal layers; score 1 represented weak cytoplasmic immunoexpression; score 2, less than 50% of cells with strong cytoplasmic staining and less than 10% with nuclear expression; and score 3, more than 50% of cells with strong cytoplasmic staining and/or more than 10% with nuclear expression. Subsequently, scores 0 and 1 were defined as low expression, and scores 2 and 3 as high expression for both proteins studied.

### Statistical analysis

The data obtained were recorded in an Excel spreadsheet (Microsoft Office 2021) and then transferred to IBM SPSS Statistics software (version 22.0; IBM Corp., Armonk, NY, USA) and STATA (version 14.0; Stata Corp., College Station, TX, USA) for descriptive and inferential statistical analysis. For all statistical tests applied in this study, the significance level was set at 5% (*p >* 0.05).

Data were subjected to the Kolmogorov–Smirnov test, which indicated a non-normal distribution. To investigate potential associations between the proteins and clinicopathological parameters of OTSCC cases, Fisher’s Exact test was applied. Spearman’s correlation coefficient (r) was applied to investigate possible correlations between protein immunoexpression scores in OTSCC cases.

To establish potential relationships between protein immunoexpression and patient prognosis, univariate and multivariate analyses were performed using 5-year Overall Survival (OS) and Disease-Free Survival (DFS). For survival analyses, the categorized values of high and low expression were considered for both markers studied. OS and DFS curves were generated using the Kaplan–Meier method and compared by the log-rank test. To identify potential prognostic factors for OS and DFS, the Cox proportional hazards model was applied. Variables with *p* < 0.20 in the log-rank test were considered candidates for inclusion in the regression analysis. Hazard ratios (HR) and 95% confidence intervals (CIs) were estimated for each variable. To minimize model instability, the multivariate model was constructed assuming at least 10 outcome events per variable.

## Results

### Sample characterization

Among the 26 cases of OTSCC, the mean age was 66.0 ± 12.9 years, ranging from 39 to 88 years. The majority of patients were male (73.1%) (male-to-female ratio 2.71:1), smokers (80.8%), and alcohol consumers (53.8%). Most tumors were classified as advanced clinical stage (III or IV) (53.8%), and 73.1% of patients were treated with surgical resection combined with radiotherapy and/or chemotherapy. Following treatment, recurrence occurred in 5 (19.2%) patients, 1 (3.8%) presented nodal metastasis, and 7 (26.9%) progressed to death, all related to OTSCC progression. Regarding histopathological grading according to WHO criteria, the majority were classified as well or moderately differentiated (46.2% each), and 65.4% presented a high-risk grading according to BD model (Table S1; Figure S1 and S2).

### Immunohistochemical study

**Fig. 1:**
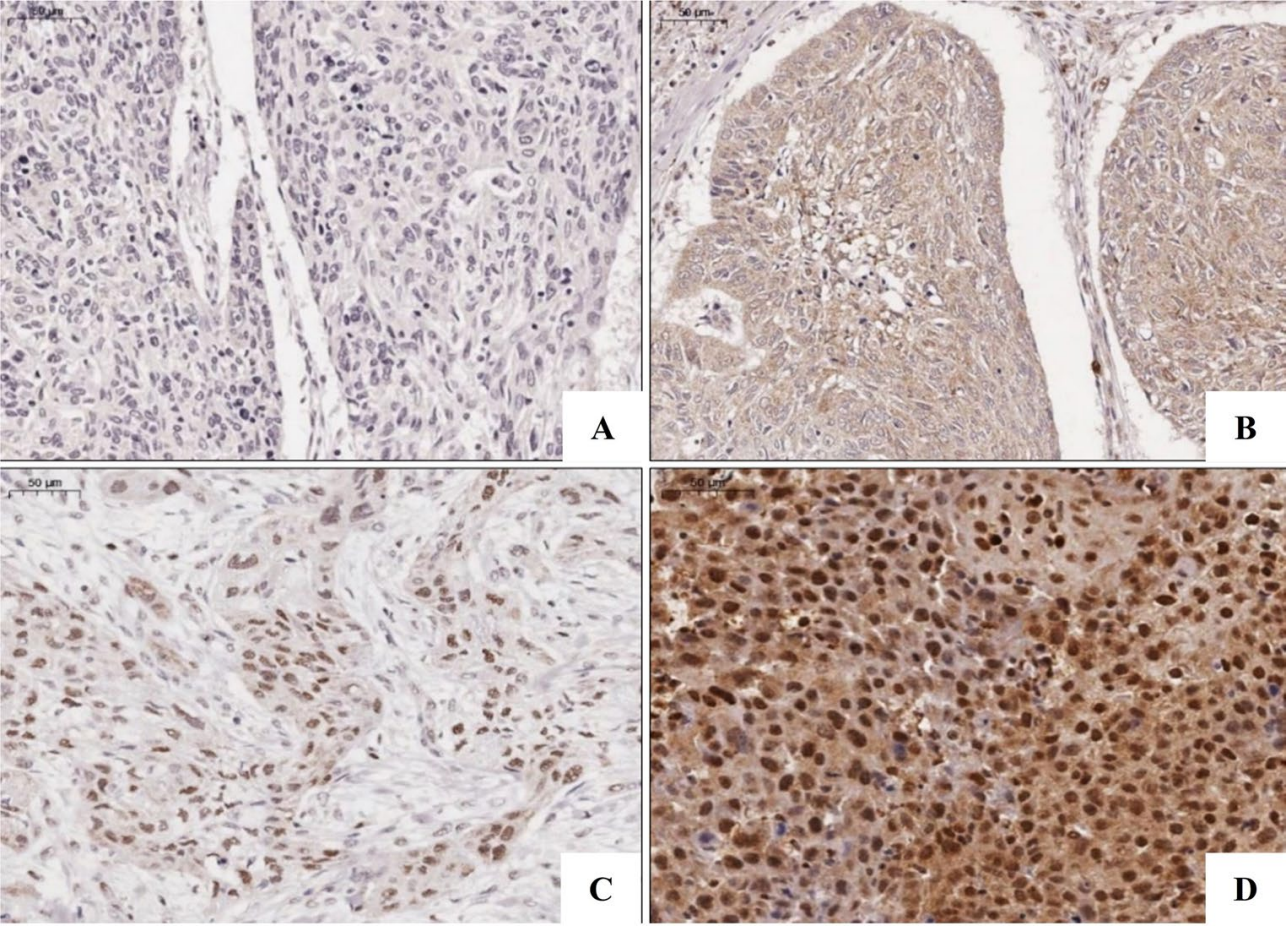
LATS2 Immunoexpression in OTSCC. (**A**) Absence of staining in neoplastic cells of some cases (Score 0). (**B**) Tumor cells exhibiting weak cytoplasmic staining (Score 1). (**C**) High nuclear expression in tumor cells (Score 3). (**D**) High nuclear and cytoplasmic immunoexpression in tumor cells, with strong nuclear staining intensity (Score 3). (Scale bar − 50 μm). OTSCC, oral tongue squamous cell carcinoma

**Fig. 2:**
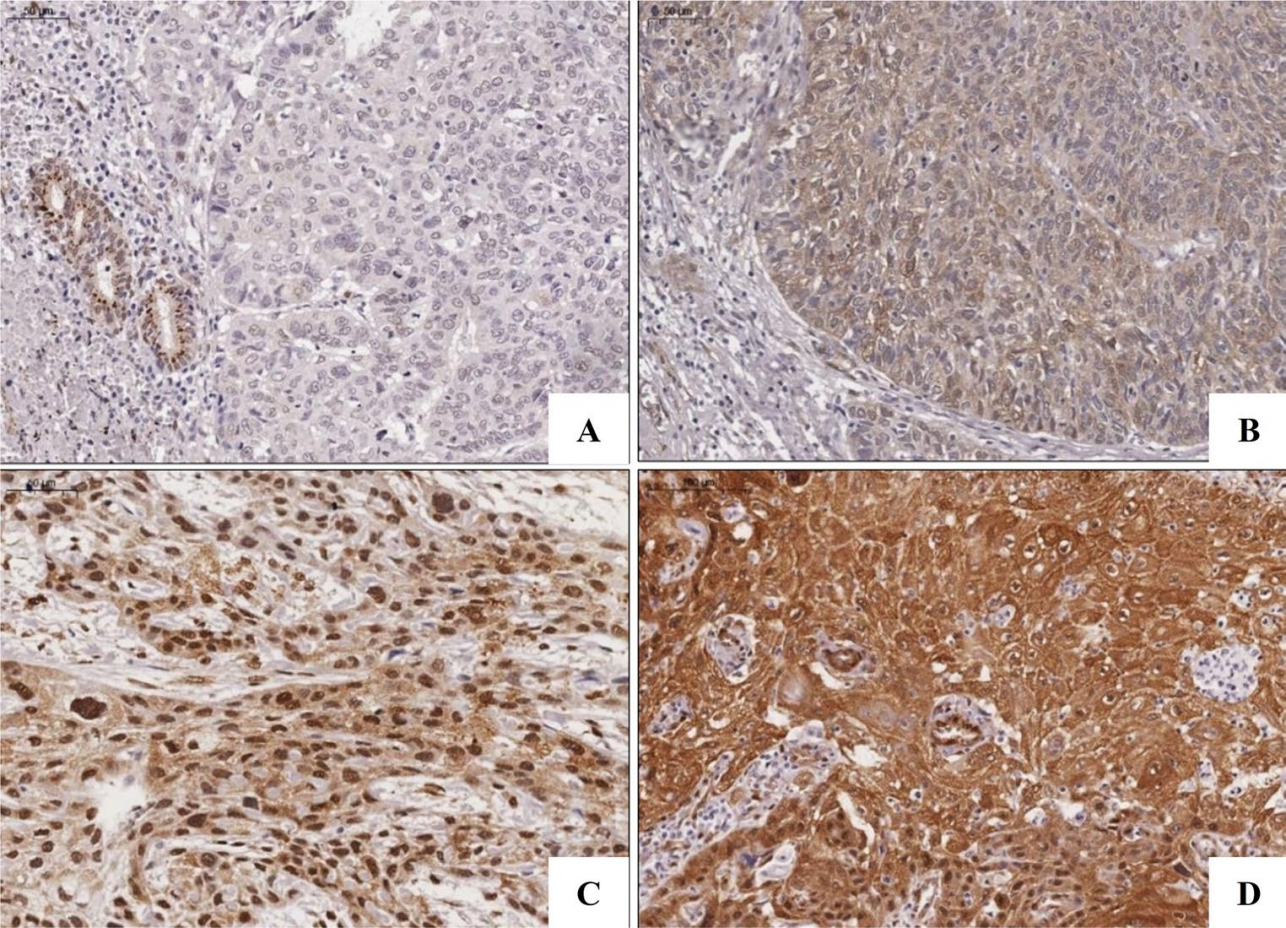
YAP1 Immunoexpression in OTSCC. (**A**) Absence of staining observed in neoplastic cells of only one case (Score 0). Adjacent to the tumor, cells of salivary gland ducts exhibiting nuclear expression. (**B**) Weak cytoplasmic staining was observed in some cases (Score 1). (**C**) Most cases exhibited high nuclear and cytoplasmic positivity (Score 3). (**D**) Predominantly strong cytoplasmic immunoexpression is also observed (Score 3). (Scale bar − 50 μm). OTSCC, oral tongue squamous cell carcinoma

LATS2 immunoexpression was observed in all epithelial layers of NOM, with a predominance of nuclear staining, while YAP1 expression was observed mainly in the basal and parabasal layers, with nuclear and cytoplasmic staining (Figure S3). In OTSCC cases, LATS2 and YAP1 showed cytoplasmic and/or nuclear staining (Figs. 1 and 2).

For LATS2, a predominance of high expression in both OTSCC (73.1%) and NOM specimens (100.0%) was observed. Regarding YAP1 expression, most OTSCC cases exhibited score 3 (high expression) (73.1%), while NOM samples were characterized by lower expression levels equally distributed between scores 0 and 1 (Tables S2). A statistically significant association was observed between high expression of YAP1 and cases diagnosed as OTSCC (*p* < 0.001). Regarding LATS2, no significant associations were observed between the studied groups (*p* > 0.05) (Table 1).

**Table 1:** Comparative analysis of LATS2 and YAP1 immunoexpression in oral tongue squamous cell carcinoma and normal oral mucosa samples

Group	LATS2				YAP1		
	Low expression	High expression	*p* ^a^		Low expression	High expression	*p* ^a^
NOM	0 (0.0)	8 (100.0)	0.160		8 (100.0)	0 (0.0)	**< **0.001*
OTSCC	7 (26.9)	19 (73.1)			5 (19.2)	21 (80.8)	

Statistically significant *p* values (< 0.05) are marked with an asterisk (*)

Abbreviations *OTSCC *oral tongue squamous cell carcimoma, *NOM *Normal Oral Mucosa

^a^Exact Fisher’s Test

Regarding the clinicopathological parameters analyzed, cases graded as high risk following BD model exhibited higher expression of YAP1 (*p* = 0.034). No additional statistically significant associations were found between the expression of the proteins studied and clinicopathological parameters (*p* > 0.05) (Table 2).

**Table 2:** Associations between the immunoexpression of LATS2 and YAP1 and clinicopathological parameters of oral tongue squamous cell carcinoma

Parameters	LATS2				YAP1		
	Low expression	High expression	*p* ^a^		Low expression	High expression	*p* ^a^
Tumor size			0.375				0.281
T1-T2	6 (33.3)	12 (66.7)			2 (11.1)	16 (88.9)	
T3-T4	1 (12.5)	7 (87.5)			3 (37.5)	5 (62.5)	
Nodal Metastasis			0.391				1.000
N (-)	5 (35.7)	9 (64.3)			3 (21.4)	11 (78.6)	
N (+)	2 (16.7)	10 (83.3)			2 (16.7)	10 (83.3)	
Clinical stage (TNM)			0.665				1.000
I-II	4 (33.3)	8 (66.7)			2 (16.7)	10 (83.3)	
III-IV	3 (21.4)	11 (78.6)			3 (21.4)	11 (78.6)	
Local recurrence			1.000				
Absent	6 (28.6)	15 (71.4)			3 (14.3)	18 (85.7)	
Present	1 (20.0)	4 (80.0)			2 (40.0)	3 (60.0)	
Second primary tumor			0.269				0.192
Absent	6 (24.0)	19 (76.0)			4 (16.0)	21 (84.0)	
Present	1 (100.0)	0 (0.0)			1 (100.0)	0 (0.0)	
Nodal metastasis after treatment			0.269				0.192
Absent	6 (24.0)	19 (76.0)			4 (16.0)	21 (84.0)	
Present	1 (100.0)	0 (0.0)			1 (100.0)	0 (0.0)	
Outcome			0.438				0.376
Remission	2 (15.4)	11 (84.6)			3 (23.1)	10 (76.9)	
Death	3 (42.9)	4 (57.1)			0 (0.0)	7 (100.0)	
WHO grading [11]			0.391				1.000
Well-differentiated	2 (16.7)	10 (83.3)			2 (16.7)	10 (83.3)	
Intermediate/ Poorly differentiated	5 (35.7)	9 (64.3)			3 (21.4)	11 (78.6)	
BD model [12]			0.661				0.034*
Low risk/Intermediate	3 (33.3)	6 (66.7)			4 (44.4)	5 (55.6)	
High risk	4 (23.5)	13 (76.5)			1 (5.9)	16 (94.1)	

Statistically significant *p* values (< 0.05) are marked with an asterisk (*)

*TNM *tumor-node-metastasis. ^a^Exact Fisher’s Test

Furthermore, when considering only OTSCC cases, a positive correlation between the immunoexpressions of LATS2 and YAP1 was evidenced, although without statistical significance (*r* = 0.246; *p* = 0.225).

### Survival analysis

For the survival analysis of patients with OTSCC, OS and DFS during 5-year period were considered. Since all deaths were cancer-related, the DSS time coincided with OS time. Lower differentiation grade according to WHO criteria (*p* = 0.022) and low LATS2 expression (*p* = 0.042) were significantly associated with decreased 5-year DFS of the patients included (Table 3).

**Table 3:** Association of 5-year overall survival (OS) and disease-free survival (DFS) with clinicopathological parameters of oral tongue squamous cell carcinoma

Parameters	*n*	OS (95% CI)	*p* ^a^		DFS (95% CI)	*p* ^a^
Age						
≤ 60 years	9	55.56 (20.42–80.45)	0.121		55.56 (43.30–98.36)	0.752
> 60 years	17	82.35 (54.71–93.94)			58.82 (11.94–77.82)	
Sex						
Male	19	68.42 (42.79–84.39)	0.383		52.63 (28.72–71.88)	0.411
Female	7	85.71 (42.79–84.39)			71.43 (28.72–91.98)	
Smoking history^*^						
No	5	1.00 (NE)	0.277		66.67 (05.41–94.52)	0.767
Yes	20	66.67 (42.54–82.50)			52.38 (29.67–70.88)	
History of alcoholism						
No	12	1.00 (NE)	0.111		83.33 (27.31–97.47)	0.119
Yes	14	64.29 (34.33–83.31)			42.86 (17.73–66.04)	
Tumor size (T)						
T1–T2	18	77.78 (51.10–91.02)	0.442		66.67 (40.35–83.43)	0.146
T3–T4	8	62.50 (22.93–86.07)			37.50 (08.70–67.44)	
Nodal metastasis (N)						
N0	14	78.57 (47.25–92.54)	0.440		57.14 (28.40–77.97)	0.916
N1–N3	12	66.67 (33.70–85.97)			58.33 (27.01–80.09)	
TNM clinical stage						
I–II	12	83.33 (48.17–95.55)	0.230		66.67 (33.70–85.97)	0.261
III–IV	14	64.29 (34.33–83.33)			50.00 (22.86–72.21)	
WHO grading [11]						
Well-differentiated	12	83.33 (48.17–95.55)	0.290		83.33 (19,49–62,65)	0.022*
Moderately-poorly differentiated	14	64.29 (34.33–83.33)			51,41 (32,08–67,74)	
BD grading model [12]						
Low-Intermediate risk	9	88.89 (43.30–98.36)	0.183		57.14 (17.19–83.71)	0.351
High risk	17	64.71 (37.71–82.34)			57.89 (33.21–76.26)	
Treatment						
Surgery only	7	57.14 (17.19–83.71)	0.152		83.33 (48.17–95.55)	0.694
Surgery + RT and/or CT	19	78.95 (53.19–91.53)			35.71 (13.03–59.44)	
LATS2						
Low expression	7	57.14 (17.19–83.71)	0.152		28.57 (04.11–61.11)	0.042
High expression	19	78.95 (53.19–91.53)			68.42 (42.79–84.39)	
YAP1						
Low expression	5	1.00 (NE)	0.160		40.00 (05.20–75.28)	0.476
High expression	21	66.67 (42.54–82.50)			61.90 (38.08–78.80)	

^a^Log-rank test. ^*^Data not available in 1 case. *TNM *tumor-node-metastasis, *RT *radiotherapy, *CT *chemotherapy, EMT epithelial-mesenchymal transition, *OS *Overall Survival, *DFS *Disease-Free Survival, *CI *Confidence Interval, *NE *Not evaluated, as the event death did not occur in one category. Statistically significant *p-*values (< 0.05) are marked with an asterisk (*)

**Fig. 3:**
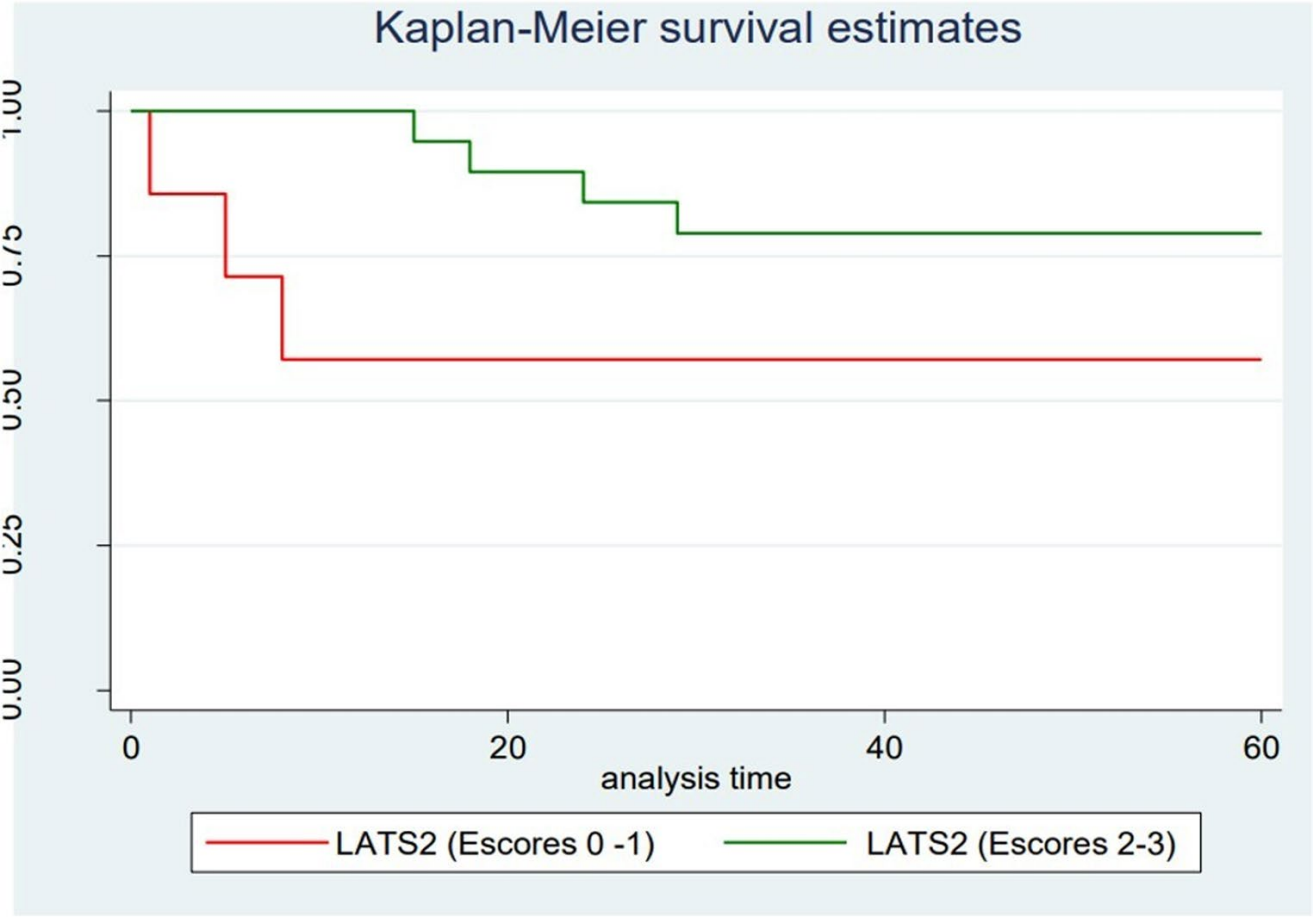
Kaplan-Meier curves for DFS with *p* values obtained by multivariate analysis models in patients diagnosed with OTSCC. (**A**) Final immunoexpression of LATS2. (**B**) Tumor size. DFS, disease-free survival; OTSCC, oral tongue squamous cell carcinoma

The multivariate analysis of OTSCC cases identified low LATS2 expression (*p =* 0.017; HRa 0.19; 95% CI 0.05–0.75) and tumor size (*p* = 0.046; HRa 3.93; 95% CI 1.02–15.06) as independent prognostic factors for 5-year DFS in OTSCC patients (Fig. 3; Table 4).

**Table 4:** Cox proportional hazards model for multivariate analysis of 5-year disease-free survival in relation to clinicopathological parameters of oral tongue squamous cell carcinoma

Parameters	HR (95% CI)	HRa (95% CI)	*p*
LATS (Low expression)	0.31 (0.09–1.02)	0.19 (0.05–0.75)	0.017*
Tumor size (T3-T4)	2.36 (0.71–7.83)	3.93 (1.02–15.06)	0.046*

*HR *Hazard Ratio, *HRa *Adjusted Hazard Ratio, *CI *Confidence Interval

Statistically significant p values ​​(≤ 0.05) are marked with an asterisk (*)

## Discussion

Advances in the identification of molecular biomarkers may enable a better understanding of the behavior of OSCC, with the potential to enhance individualized therapeutic modalities and favor better prognoses for these patients [14]. Some studies have been evaluating the participation of Hippo pathway proteins in the process of carcinogenesis in various human tumors [9, 15]. However, investigations assessing LATS2 and YAP1 proteins in OSCC are still scarce. To date, this is the first research to evaluate the immunohistochemical expression of LATS2 and its associations with clinicopathological parameters and survival of patients with OSCC.

In this study, high expression of LATS2 was observed in NOM and in most OTSCC cases. These findings partially corroborate those described by Zhang et al. (2010), who reported high LATS2 expression in nasopharyngeal carcinomas and low expression of this protein in normal nasopharyngeal epithelium [16]. However, Han, Yin, Zhang (2018) [17] observed that LATS2 immunoexpression was significantly lower in hepatocellular carcinomas when compared to normal tissue samples. In turn, Zhao et al. (2021) identified low staining of this protein in 66.2% of colorectal cancer cases [18]. In the present study, approximately a third of OTSCC cases exhibited low expression for LATS2 ]. Therefore, it is suggested that the expression of this protein may vary depending on the type of cancer analyzed, as well as within the same type of tumor. This may occur due to inter-tumoral and intra-tumoral heterogeneity, illustrated by the genetic and phenotypic diversity that malignant cells can exhibit within the same cancer, as well as intrinsic and extrinsic conditions to the tumor, which can reflect the molecular profile of each case [19, 20].

For YAP1, in NOMs, there was nuclear immunopositivity restricted to the basal and parabasal layers, in addition to weak cytoplasmic staining. The expression of this protein was significantly higher in OTSCC cases (nuclear and cytoplasmic). These data were also described by Ono et al. (2019) [13]. Omori et al. (2020) identified a gradual increase in YAP1 expression in NOMs, oral epithelial dysplasia, and OTSCC, with the most invasive carcinomas showing the most intense staining for this protein [21]. Our data indicate a potential association between YAP1 activation and the progression of OTSCC.

In the present study, lower LATS2 expression was associated with poor DFS both in univariate and multivariate analysis, consistent with the findings of Matsuda et al. (2021), who identified a poor DFS rate in prostate carcinomas with lower LATS2 expression [22]. The impact of the low expression of this protein in prognosis was also evidenced by Jang et al. (2019) in patients with small cell lung carcinoma [23]. As one of the main counter-regulators of YAP1, low LATS2 expression can lead to greater YAP1 translocation to the nucleus. These higher levels of nuclear YAP1, in turn, can regulate the transcription of several genes involved in cell proliferation, migration, and survival, thus contributing to poor prognoses, as already described previously [9, 24, 25].

Concerning clinicopathological parameters, better DFS was identified in well-differentiated OTSCC cases according to WHO criteria. Although the relationship between conventional histological grading and clinical outcome is well-established, this system has been considered controversial. Moreover, cases graded as high-risk according to BD model grading system exhibited higher YAP1 scores. The BD model evaluates the effectiveness of tumor penetration into tissues through the depth of tumor invasion, as well as tumor budding and its dissociation power at the tumor front, which is considered an important factor in the epithelial-mesenchymal transition (EMT) [26].

The cell tumors hability of migration and invasion are important events in EMT and appear to be influenced by YAP1/TAZ activity [25, 27]. Li et al. (2020) reported that two invasion-associated proteins, MMP2 and MMP9, had their expression reduced after YAP1 silencing in head and neck squamous cell carcinoma cells [28]. In turn, Ahmad, Parkinson, Wan, (2022) evidenced that high levels of desmoglein-3 (DSG-3), an important molecule in cell adhesion and tissue integrity, negatively regulate the nuclear activity of YAP1 and increase its cytoplasmic expression due to the cytoplasmic translocation, which may restrict the capacity for cell migration in OSCC cell lines [24]. These authors also highlight that high YAP1 levels may not be indicative of its activity, given that the transcriptional activity of YAP1 is predominantly associated with its nuclear localization. Therefore, considering that our study identified high YAP1 expression in the nucleus of neoplastic cells, it is suggested that nuclear YAP1 expression may be associated with EMT and cell invasion in OTSCC.

No significant associations were found between YAP1 immunostaining and the evaluated survival rates. These findings disagree with the results evidenced by Omori et al. (2020), who verified associations between high YAP1 expression (nuclear and cytoplasmic) and lower OS and DFS in patients with OTSCC [21]. Ono et al. (2019) also reported the association between elevated YAP1 expression (nuclear or nuclear and cytoplasmic) and a poor prognosis in patients with OSCCs [13]. Thus, YAP1 expression (either only nuclear or nuclear and cytoplasmic) appears to influence the survival of patients with OSCC.

Furthermore, YAP1 activity can be regulated, directly or indirectly, by several proteins involved in the Hippo signaling cascade other than LATS2, such as MST1/2, SAV1, MOB1, and LATS1 [9, 29]. Omori et al. (2020) showed that the deletion of MOB1 (a molecule that aids in LATS phosphorylation) in mice tongues resulted in endogenous YAP1 hyperactivation and led to rapid carcinogenesis, establishing an invasive OTSCC within four weeks [21]. Furthermore, Yang et al. (2024b) report that the expressions of LATS1 and LATS2 are not interdependent and, depending on inter- and intra-tumoral heterogeneity, they may have opposing functions [30].

In addition, YAP1 can also be regulated by interactions independent of the Hippo pathway. Among these interactions, several proteins including α-catenin, AMOT, PTPN14, and CDK1 can influence directly on YAP1 activity [31]. Rodrigo et al. (2024) revealed significant communication between YAP1 and the PI3K/mTOR pathway, evidenced by the positive correlation between YAP1 and PIK3CA [32]. The nuclear activation of YAP1 was associated with larger tumor size, lymph node metastasis, advanced stage, and poor differentiation, configuring a more aggressive phenotype. Furthermore, Sato et al. (2025) demonstrated that the loss of function or alteration in the copy number of FAT1 can result in YAP1 hyperactivation, driving oncogenesis [33]. Therefore, due to the complex nature of Hippo pathway regulation in the process of carcinogenesis, to solely determine YAP1 activity, either in isolation or dependent on a single pathway, may not completely answer the existing questions.

The dysregulation of the Hippo pathway has been associated with therapeutic resistance in various types of cancer, with the YAP1/TAZ/TEAD pathway being pointed out as a relevant target to overcome drug resistance and multi-drug therapies [34]. Peptide mimetics and small molecule inhibitors that block this pathway also demonstrate suppression of transcription and tumor growth, as is the case of Verteporfin, the first small molecule identified [34, 35]. Furthermore, other small molecules are being identified, some acting directly on TEAD, such as flufenamic acid, IK-930, and VT3989, in addition to new technologies focused on protein degradation, which aim to physically eliminate YAP1/TAZ or TEAD [34]. To date, many of these inhibitors are still being studied to better understand their anti-tumor effects and toxicity [34, 36].

One of the limitations of this study refers to the reduced sample size that met the pre-established inclusion criteria, mainly related to patient survival data. Thus, prognostic implications inferred in this investigation should be interpreted cautiously. Furthermore, the expression of the proteins evaluated in this research through immunohistochemistry allows for an excellent in situ analysis, but it does not permit the identification of specific molecular alterations that could definitively clarify how the Hippo pathway influences the pathogenesis of OTSCC.

## Conclusion

This study suggests that the functional alteration of the LATS2 and YAP1 proteins may influence the biological behavior of OTSCC. Reduced LATS2 expression was associated with lower DFS, while YAP1 expression was related to a higher risk according to the BD model. These findings indicate that the dysregulation of the Hippo pathway may represent an unfavorable prognostic factor, possibly influencing some mechanisms of tumor progression, such as EMT and cell invasion.

## Supplementary Information

Below is the link to the electronic supplementary material.

Supplementary Material 1

## Data Availability

The data that supports the findings of this study is available from the corresponding author upon reasonable request.

## Declarations

**Ethics statement**.

This study was approved by the Research Ethics Committee of the Federal University of Rio Grande do Norte (Approval No. 5,288,936).
