# Supplementary material for: Immunoexpression profile of LATS2 and YAP1 and its clinicopathological relevance in oral tongue squamous cell carcinoma
Source: Oral Maxillofac Surg. 2026 Apr 14;30(1):70. doi: 10.1007/s10006-026-01557-1 (PMC13079482; doi:10.1007/s10006-026-01557-1)
Supplement: Supplementary file 1 — Supplementary Material 1 [file 10006_2026_1557_MOESM1_ESM.docx]

***Title:*** IMMUNOEXPRESSION PROFILE OF LATS2 AND YAP1 AND ITS CLINICOPATHOLOGICAL RELEVANCE IN TONGUE SQUAMOUS CELL CARCINOMA

***Journal name*:** Oral and Maxillofacial Surgery

***Author:*** Ondina Karla Mousinho da Silva Rocha^a^, PhD (ondina_rocha@hotmail.com) - 0000-0002-7328-0934,

André Luis Alves Borges^a^, MSc (andrelaborgezp@gmail.com) - 0000-0003-3174-8882,

Lucas Melo da Costa^a^, MSc (lucas-mdc@hotmail.com ) - 0000-0003-3321-3689,

Débora Frota Colares^a^, MSc (deborafrotac@live.com) - 0000-0002-8787-5904,

Éricka Janine Dantas da Silveira^b^, PhD (ericka_janine@yahoo.com.br) - 0000-0003-2128 0147,

Márcia Cristina da Costa Miguel^b^, PhD(mccmiguel@hotmail.com) - 0000-0002-6661-2566.

***^a^****Postgraduate Program in Dental Sciences, Department of Oral Pathology, Federal University of Rio Grande do Norte, Natal, Rio Grande do Norte, Brazil.*

***^b^****Department of Oral Pathology, Federal University of Rio Grande do Norte, Natal, Rio Grande do Norte, Brazil*.

***Corresponding Author:***

Márcia Cristina da Costa Miguel

Department of Oral Pathology, Federal University of Rio Grande do Norte, Natal, Rio Grande do Norte, Brazil.

Address: Av. Salgado Filho, 1787, Lagoa Nova – Natal/RN. Zip code: 59056-000

Telephone: 55 84 3215-4138

E-mail: [mccmiguel@hotmail.com](mailto:mccmiguel@hotmail.com)


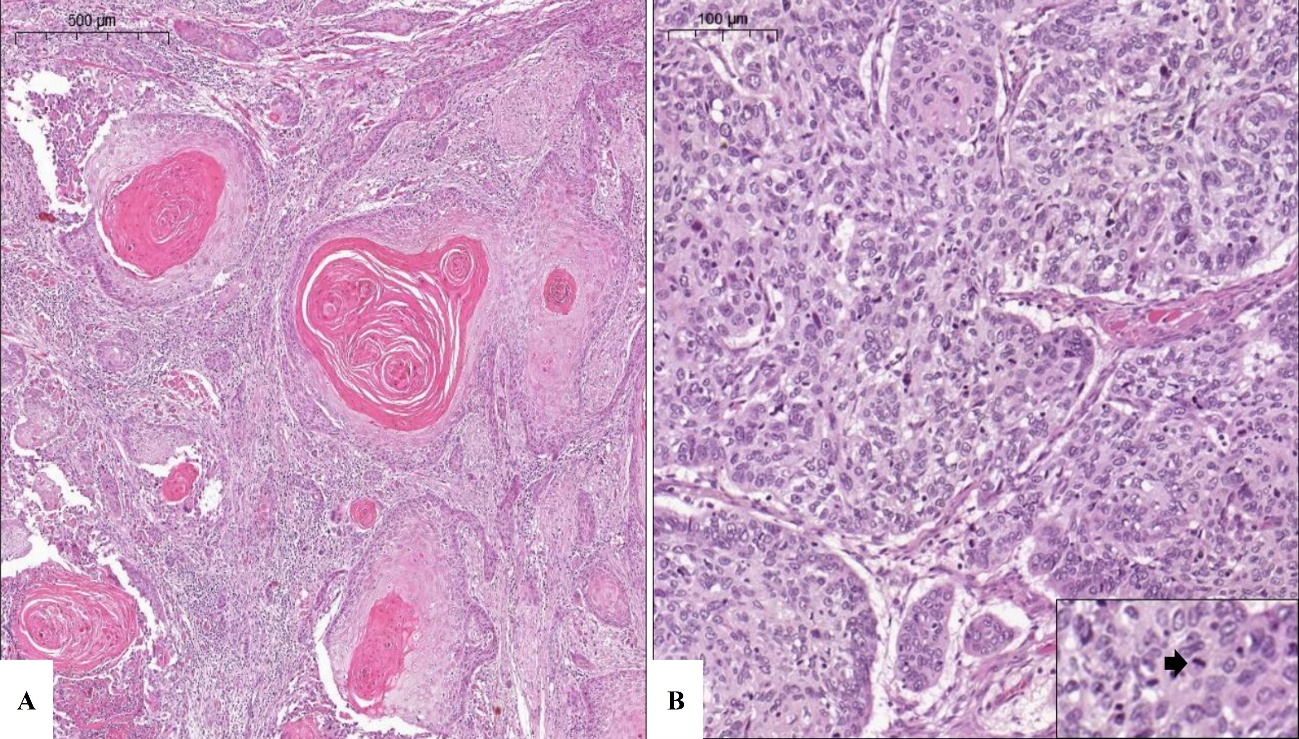
**Supplementary Fig.1 Morphological aspects of OTSCC according to the WHO grading system (H&E)**. **(A)** Well-differentiated OTSCC exhibiting tissue architecture similar to the epithelium of origin with numerous keratin pearls. **(B)** Poorly differentiated OTSCC showing immature cells, absence of keratinization, and mitotic figures (black arrow). (Scale bar, 100μm - 500μm). OTSCC, oral tongue squamous cell carcinoma.


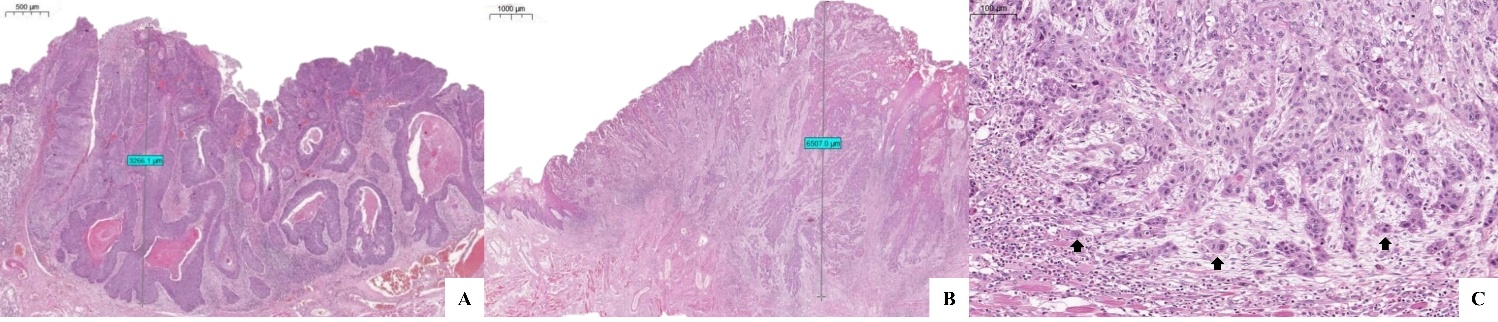
**Supplementary Fig.2 OTSCC grading according to BD model (H&E). (A)** Invasion depth shorter than 4 mm, with tumor nests containing more than 5 cells at the invasive front. **(B)** Invasion depth greater than 4 mm. **(C)** Presence of tumor buds, each with fewer than 5 cells, at the invasive front (black arrows). (Scale bar, 100μm - 500μm). OTSCC, oral tongue squamous cell carcinoma.

**Supplementary Fig.3 S3 Immunoexpression of LATS2 and YAP1 in NOM. (A)** LATS2 expression was observed throughout all epithelial layers, with predominantly nuclear staining. (Score 3) **(B)** YAP expression demonstrating nuclear and cytoplasmic staining confined to the basal layer (Score 0). (Scale bar, 100μm). OTSCC, oral tongue squamous cell carcinoma; NOM, normal oral mucosa.


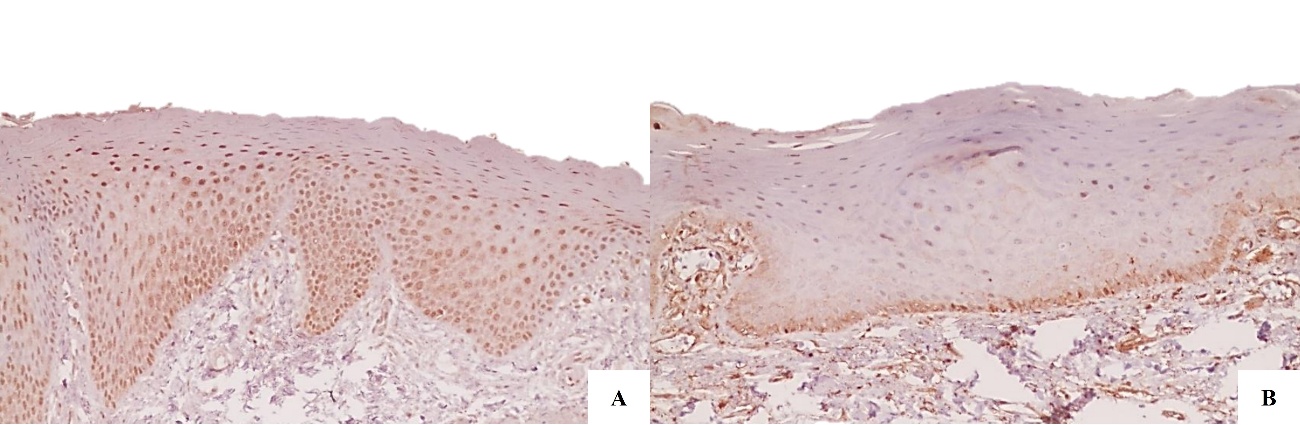


**Supplementary Table 1.** Absolute and relative distribution of oral tongue squamous cell carcinoma cases according to clinicopathological parameters.

| **Parameters** | ***n* (%)** |
| --- | --- |
| **Age** |  |
| ≤ 40 years | 1 (3.8) |
| 41–60 years | 8 (30.8) |
| > 60 years | 17 (65.4) |
| **Sex** |  |
| Male | 19 (73.1) |
| Female | 7 (26.9) |
| **Smoking history** |  |
| Yes | 20 (76.9) |
| No | 5 (19.2) |
| No information | 1 (3.8) |
| **History of alcoholism** |  |
| Yes | 12 (42.6) |
| No | 14 (53.8) |
| **Tumor size (T)** |  |
| T1 | 5 (19.2) |
| T2 | 13 (50.0) |
| T3 | 5 (19.2) |
| T4 | 3 (11.5) |
| **Nodal metastasis (N)** |  |
| N0 | 14 (53.8) |
| N1 | 7 (26.9) |
| N2 | 5 (19.2) |
| **Distant metastasis (M)** |  |
| M0 | 26 (100.0) |
| M1 | 0 (0.0) |
| **TNM clinical stage** |  |
| I – II | 12 (46.2) |
| III – IV | 14 (53.8) |
| **WHO grading [11]** |  |
| Well differentiated | 12 (46.2) |
| Moderately differentiated | 12 (46.2) |
| Poorly differentiated | 2 (7.7) |
| **BD grading model [12]** |  |
| Low Risk | 3 (11.5) |
| Intermediate A | 4 (15.4) |
| Intermediate B | 2 (7.7) |
| High Risk | 17 (65.4) |
| **Treatment** |  |
| Surgery only | 7 (26.9) |
| Surgery + RT/ CT | 19 (73.1) |
| **Second Primary tumor** |  |
| Absent | 25 (96.2) |
| Present | 1 (2.8) |
| **Nodal metastasis (after starting treatment)** |  |
| N0 | 25 (96.2) |
| N+ | 1 (3.8) |
| **Distant metastasis (after starting treatment)** |  |
| M0 | 26 (100.0) |
| M1 | 0 (0.0) |
| **Local recurrence** |  |
| No | 21 (80.8) |
| Yes | 5 (19.2) |
| **Clinical outcome** |  |
| Remission | 13 (50.0) |
| Alive with disease | 6 (23.1) |
| Death | 7 (26.9) |
| **Total** | 26 (100.0) |

TNM, tumor-node-metastasis; RT, radiotherapy; CT, chemotherapy.

**Supplementary Table 2**. Absolute and relative distribution of oral tongue squamous cell carcinoma and normal oral mucosa samples according to the immunoexpression scores of LATS2 and YAP1.

| **Biomarkers** | **OTSCC** | | | |  | **NOM** | | | |
| --- | --- | --- | --- | --- | --- | --- | --- | --- | --- |
|  | **Score 0**  ***n* (%)** | **Score 1**  ***n* (%)** | **Score 2**  ***n* (%)** | **Score 3**  ***n* (%)** |  | **Score 0**  ***n* (%)** | **Score 1**  ***n* (%)** | **Score 2**  ***n* (%)** | **Score 3**  ***n* (%)** |
| **LATS2** | 4 (15.4) | 3 (11.5) | 4 (15.4) | 15 (57.7) |  | 0 (0.0) | 0 (0.0) | 0 (0.0) | 8 (100.0) |
| **YAP1** | 1 (3.8) | 4 (15.4) | 2 (7.7) | 19 (73.1) |  | 4 (50.0) | 4 (50.0) | 0 (0.0) | 0 (0.0) |
